# Visibility Is Not Control: Construct Separation and Variable Attribution in Surgical Reimbursement Policy

**DOI:** 10.7759/cureus.113000

**Published:** 2026-07-20

**Authors:** Andrew M Klapper, Anthony N Dardano, Michael Risin, Karla Maita, Monali Mahedia

**Affiliations:** 1 Plastic and Reconstructive Surgery, Florida Atlantic University Charles E. Schmidt College of Medicine, Boca Raton, USA; 2 Plastic and Reconstructive Surgery, Delray Medical Center, Delray Beach, USA; 3 Plastic Surgery, Mayo Clinic, Jacksonville, USA; 4 Plastic Surgery, Larkin Community Hospital, Miami, USA

**Keywords:** construct separation, health policy, independent dispute resolution, network adequacy, no surprises act, physician reimbursement, qualifying payment amount, specialist availability, surgical reimbursement, variable attribution

## Abstract

Surgical reimbursement policy frequently treats dollar-denominated figures as interchangeable even when they measure different economic constructs. Physician professional fees, facility revenue, Medicare Physician Fee Schedule rates, qualifying payment amounts, contracted rates, billed charges, adjudicated payments, and total episode costs differ in provenance, construction method, purpose, and interpretive boundary. A related attribution error occurs when accountability is assigned to the actor most visible to the patient rather than to the actor that controlled the operative variable. This technical report proposes a construct-separation and variable-attribution framework for distinguishing what reimbursement figures measure from who controlled the variables that produced disputed payment, access, or cost-containment outcomes. The report presents two operational checks: a five-filter construct-separation test and a six-question variable-attribution test. These checks apply symmetrically to physician charges, payer benchmarks, adjudicated outcomes, directory-based network claims, and savings assertions. Functional specialist availability is identified as an access-relevant construct distinct from directory enrollment. A network listing may establish contractual presence but does not establish emergency coverage, complex case acceptance, transfer acceptance, after-hours availability, or post-operative continuity. The framework does not set prices, validate billed charges, invalidate statutory benchmarks, or prove access harm. It establishes a measurement discipline that should precede policy judgment: identify the construct, define its boundary, state what it omits, and map operative control before assigning accountability. The intended audience includes clinicians, health-policy researchers, payers, regulators, hospitals, independent dispute-resolution entities, and legislators seeking a structured method for interpreting reimbursement figures, evaluating network adequacy, and assigning accountability according to operative control rather than patient-facing visibility.

## Introduction

Healthcare cost policy often begins with a number and proceeds too quickly to a conclusion. A payment amount may be described as high, low, reasonable, excessive, market-based, statutory, or cost-saving before the analysis has established what the number actually measures. That shortcut is the core methodological error addressed in this technical report.

In the United States, these interpretive problems are especially visible under the No Surprises Act (NSA), which created a federal process for resolving certain out-of-network payment disputes. The qualifying payment amount (QPA), generally based on an insurer’s median contracted rate for a service in a geographic region, serves as an important statutory reference point. When the parties cannot agree on payment, the dispute may proceed to federal independent dispute resolution (IDR), where a certified entity considers the parties’ offers and the permitted statutory information. The Medicare Physician Fee Schedule (MPFS) represents a separate federal administrative payment system based on relative value units, a conversion factor, geographic adjustments, and statutory policy constraints. Although QPAs, MPFS rates, contracted rates, billed charges, and adjudicated payments are all expressed in dollars, they were created for different purposes and should not be treated as interchangeable measures of value, complexity, or market price.

Consider a simplified reimbursement dispute. A surgeon performs an emergency reconstruction and submits a billed charge. The payer cites a QPA or MPFS rate as evidence that the charge is excessive. The patient sees the physician’s bill and may conclude that the physician caused the financial dispute. Yet the billed charge, QPA, and MPFS rate are different economic constructs, and the physician may not have controlled the network design, benchmark methodology, claims-processing sequence, or payment offer that produced the dispute. This example illustrates the two errors addressed in this report: construct drift, in which one reimbursement figure is used as a proxy for another, and attribution drift, in which accountability is assigned to the most visible actor rather than to the actor controlling the operative variable.

Dollar-denominated figures invite comparison because they share a common unit, even when they do not share a common construct. Physician professional fees, facility revenue, MPFS rates, QPAs, contracted rates, billed charges, adjudicated payments, and downstream episode costs are different economic objects. Each has a different source, construction method, purpose, and boundary. Using one as a proxy for another creates construct drift: the number remains precise, but the inference becomes unstable [1-5].

A second error is attribution drift. Once a dispute becomes visible, responsibility tends to attach to the actor closest to the patient. The physician appears on the bill, performs the service, and explains the problem. Yet the physician may not have controlled network architecture, benchmark construction, authorization rules, claims-processing sequence, statutory timelines, or directory accuracy. Physicians also control clinically material variables, including treatment selection, documentation, code assignment, case acceptance, and timing. Accurate analysis requires variable attribution, not default blame in either direction [5,6].

Visibility identifies where the patient encounters the problem; it does not identify who controlled it. This distinction is particularly important in network-adequacy analysis, where a directory listing answers a contractual-enrollment question but does not establish whether a specialist is available for emergency care, complex transfers, salvage cases, after-hours coverage, or post-operative continuity [6-9].

Existing reimbursement-policy approaches commonly examine payment benchmarks, network adequacy, administrative burden, professional reimbursement, or total episode cost as separate domains. The proposed framework differs by requiring two sequential determinations before a policy inference is made: first, whether the cited number is being used within the boundaries of the economic construct it actually measures; and second, whether accountability has been assigned to the actor who controlled the operative variable rather than simply to the actor most visible to the patient. To our knowledge, construct separation and variable attribution have not previously been combined into a single operational framework for reimbursement, access, and accountability analysis.

This report proposes a two-part framework with two named operational tests. Construct separation, implemented through a five-filter test, asks what economic object is being measured. Variable attribution, implemented through a six-question test, asks which actor controlled the variable that produced the disputed outcome. A third contribution, functional specialist availability, distinguishes the operational access question that matters for patient care from the directory-enrollment measure that often dominates network-adequacy analysis.

## Technical report

Methods and framework purpose

This article is a narrative health-policy technical report and conceptual policy-analysis framework. It does not involve human subjects, identifiable patient data, clinical intervention, institution-specific claims data, proprietary payer data, or quantitative claims modeling. No institutional review board approval was required because the report analyzes publicly available policy documents, published literature, and conceptual reimbursement scenarios rather than patient-level data.

The literature and policy review was targeted rather than systematic. Sources were identified through focused searches of PubMed, federal agency websites, statutory and regulatory materials, and reference lists of relevant policy publications. Search concepts included "physician reimbursement", "Medicare Physician Fee Schedule", "qualifying payment amount", "No Surprises Act", "independent dispute resolution", "national health expenditures", "administrative costs", "physician consolidation", "network adequacy", "specialist availability", and "total episode cost". Priority was given to primary federal sources, peer-reviewed studies, current regulations, national expenditure datasets, and publications directly relevant to the framework’s core domains. Sources were included when they clarified the construction, purpose, or interpretive limits of a reimbursement or access measure. The review was not designed to capture all available literature, assess study quality systematically, or perform quantitative synthesis.

The analytic approach proceeded in three steps. First, the report classified commonly cited reimbursement figures according to source, construction method, intended purpose, and interpretive boundary. Second, the report translated those distinctions into a five-filter construct-separation test and a six-question variable-attribution test. Third, illustrative policy examples were used to show how the framework applies to physician charges, facility fees, MPFS rates, QPAs, billed charges, adjudicated payments, total episode cost, directory-based network claims, and functional specialist availability [1-15].

Unless otherwise identified as cited empirical findings, the reimbursement disputes, clinical scenarios, control maps, and policy examples presented throughout this report are hypothetical and illustrative. They were constructed solely to demonstrate application of the proposed framework and were not derived from identifiable patients, actual IDR disputes, proprietary claims data, or institution-specific reimbursement cases.

Illustrative application of the framework

To demonstrate practical application, consider a realistic hypothetical emergency reconstructive case in which a surgeon submits a billed charge of $20,000, the payer cites an MPFS amount of $2,500, the QPA is $4,000, and an independent dispute-resolution entity ultimately selects a payment of $9,000. These amounts are intentionally simplified and are not derived from a patient case.

The five-filter construct-separation test first identifies each amount as a different economic object. The billed charge is the provider’s requested amount; the MPFS amount is an administratively determined Medicare payment; the QPA is an issuer-calculated median contracted-rate benchmark; and the adjudicated amount is the outcome of a specific statutory dispute process. The framework does not treat any of these figures as automatically establishing market value or appropriate payment.

The six-question variable-attribution test then maps control. The physician controlled treatment selection, documentation, coding, case acceptance, and the submitted charge. The payer controlled network design, QPA construction, initial payment strategy, and claims processing. The IDR entity controlled the final offer selection within the statutory framework. The patient primarily encountered the physician’s bill, but patient-facing visibility did not establish control over the benchmark, network configuration, or adjudication process.

The framework does not determine that $9,000 was correct, that $20,000 was justified, or that the payer’s benchmark was inadequate. Its function is narrower: to prevent a billed charge, administrative fee schedule, contractual benchmark, and adjudicated payment from being treated as interchangeable evidence and to prevent accountability from being assigned solely to the actor most visible to the patient. Although the numerical values are hypothetical, the payment constructs and allocation of control reflect commonly encountered features of United States reimbursement disputes. No statistical methods, formal pilot testing, inter-rater assessment, or independent empirical validation were performed in this conceptual report. The five-filter construct-separation test and six-question variable-attribution test were refined through iterative author review and application to hypothetical reimbursement and access scenarios. This internal development process was used to improve clarity and practical usability but did not constitute independent expert validation, formal pilot testing, or reproducibility assessment. Throughout the report, cited expenditure figures, statutory provisions, and published access measures are treated as evidence-supported observations, whereas the proposed tests, construct classifications, control maps, and policy applications are the authors’ conceptual interpretations.

Construct separation: what is being measured?

A defensible reimbursement analysis begins by identifying the construct being measured. Physician professional fees, facility fees, MPFS rates, commercial contracted rates, QPAs, billed charges, adjudicated payments, and total episode costs are not interchangeable proxies for one another. Each has a source, purpose, and interpretive boundary. Payment constructs should not be treated as interchangeable merely because they are expressed in the same unit.

Table 1 compares common payment constructs and highlights their intended measurement domain, important interpretive boundaries, and examples of inference errors that may occur when a construct is used beyond its original purpose. These examples support construct-separation analysis rather than establish payment adequacy, market value, or reimbursement appropriateness.

**Table 1:** Payment constructs: what each measures, what it does not, and the misuse risk. MPFS = Medicare Physician Fee Schedule; RVU = relative value unit; NSA = No Surprises Act; QPA = qualifying payment amount.

Construct	What It Measures	What It Does Not Measure	Misuse Risk
Physician professional fee	Clinical work, judgment, procedural skill, liability exposure, availability, follow-up, and practice infrastructure.	Facility overhead, insurer administration, pharmaceutical spending, or total episode cost.	Treated as equivalent to total medical spending for an encounter.
Facility fee	Site infrastructure, nursing, equipment, space, and facility overhead.	Physician professional work unless separately enumerated.	Professional fee obscured within larger encounter cost.
MPFS rate	Medicare payment under a federal administrative fee schedule using RVUs, conversion factor, and geographic adjustors.	Commercial market value, case complexity, or out-of-network adequacy.	Used as neutral market anchor despite statutory and budgetary constraints.
Commercial contracted rate	Network-specific negotiated payment for a defined service.	Universal fair value or complete clinical complexity.	Treated as general market value despite leverage and network effects.
QPA	Issuer-calculated median contracted-rate benchmark under NSA methodology.	Independent market-clearing price, clinical value, downstream cost, or case-specific complexity.	Treated as self-validating fair-value indicator.
Billed charge	Provider's stated requested amount.	Reasonable payment expectation in isolation.	Treated as value without contextual validation.
Adjudicated payment	Case-specific dispute-resolution outcome after statutory factors are considered.	Universal payment entitlement or market-wide benchmark.	Overgeneralized beyond selected completed disputes.
Total episode cost	Aggregate downstream spending across a care trajectory.	Professional-fee adequacy unless separately decomposed.	Used without identifying contributing cost drivers.

Denominator discipline and administrative benchmarks

Construct separation requires denominator discipline: a sector cannot be assigned responsibility for cost growth unless the numerator and denominator are correctly defined. The CMS physician-and-clinical-services category accounted for approximately 21% of national health expenditures in 2024 [1,3], but this category is broader than physician professional fees alone because it includes outpatient care centers and independently billing medical and diagnostic laboratory services. A narrower Census / FRED measure of physician-office gross revenue was approximately $601 billion in 2022, or roughly 13% of national health expenditures that year [2]. Neither figure represents physician take-home income; both reflect gross practice revenue that supports staffing, malpractice coverage, compliance infrastructure, call coverage, and clinical availability before any physician compensation is realized. These figures reflect the most recent directly comparable national datasets identified for each construct at the time of manuscript preparation; the difference in reference years reflects source-specific reporting availability rather than an attempt to compare contemporaneous measures.

A simplified numerical example illustrates why denominator discipline matters. If a policy analysis states that physician-and-clinical-services spending represents approximately 21% of national health expenditures, that figure cannot be interpreted as the physician professional-fee share because the category includes additional outpatient and diagnostic services. Conversely, if physician-office gross revenue represents approximately 13% of national health expenditures, that percentage still does not represent physician compensation because gross revenue includes practice overhead and infrastructure. The same dollar figure may therefore support very different conclusions depending on whether the denominator is national spending, total episode cost, gross practice revenue, or net professional compensation. These numerical values are descriptive examples drawn from cited national datasets; the interpretive distinction is conceptual rather than statistically tested.

MPFS rates administer Medicare payment through a federal fee schedule governed by relative value units, conversion-factor policy, budget-neutrality constraints, and statutory update rules; they were not designed to measure commercial market value or case-specific clinical complexity outside Medicare payment administration [10]. QPA is an issuer-calculated median contracted-rate benchmark under the NSA. It is legally relevant and operationally important, but its legal relevance does not make it economically complete for questions about case-specific clinical complexity, downstream failure risk, functional specialist availability, or total episode cost [11].

Code equivalence and total episode cost

Coding systems identify services; they do not fully capture the clinical environment in which those services are performed. The same code may describe a controlled elective procedure or an emergency intervention in a contaminated wound, irradiated field, ischemic limb, anticoagulated trauma patient, infected prosthetic region, exposed hardware environment, failed prior closure, pediatric injury, geriatric skin-tear avulsion, or multiply operated scarred field.

Table 2 summarizes selected clinical complexity variables that may not be fully captured by code-only comparisons. These variables do not invalidate coding systems or reimbursement benchmarks; rather, they illustrate circumstances in which procedural equivalence may not imply clinical equivalence. The variables listed in Table 2 are derived from common clinical scenarios described in surgical practice and published literature, but the table itself is conceptual and was not generated from a patient cohort or quantitative dataset.

**Table 2:** Clinical complexity variables that may not be fully captured by code-only comparison. Examples are illustrative rather than exhaustive and are intended to support construct-separation analysis rather than establish payment adequacy.

Variable	Why It Matters	Benchmark Risk
Urgency / emergency setting	Limits planning, staffing, time, and alternatives.	Elective benchmark may be applied to non-elective care.
Contamination / infection	Increases failure risk and aftercare burden.	Code may not represent the biological risk environment.
Hardware or prosthesis exposure	Raises stakes of failure and salvage complexity.	Benchmark may not capture downstream exposure risk.
Ischemia, radiation, or poor tissue quality	Reduces healing reserve and increases failure probability.	Procedure label may understate technical difficulty.
Prior failure or revision field	Scar and altered anatomy increase complexity.	Code may treat primary and salvage cases similarly.
Geriatric or pediatric fragility	Changes tissue handling and tolerance for failure.	Benchmark may not capture population-specific vulnerability.
Call burden and emergency availability	Reflects standby capacity and liability exposure.	Network participation may not equal functional clinical availability.

In complex surgical care, the index procedure may represent only one part of the economic consequence of treatment selection. A visible price reduction is not equivalent to system savings unless downstream utilization, site-of-service migration, and access effects are also measured. Conceptual frameworks for distinguishing index procedural cost from downstream total episode expenditure in complex wound reconstruction have described the cost consequences of treatment selection across the care trajectory [12].

Variable attribution: who controlled the operative variable?

Construct separation establishes what type of economic object is being cited. A second analytic question follows: which actor or structure controlled the variable being interpreted? In policy analysis, accountability should follow control of the operative variable. Visibility identifies where the patient experiences the problem; it does not establish who produced it.

Variable attribution is bidirectional. Physicians control treatment selection, documentation quality, code assignment, timing of intervention, case acceptance, and willingness to manage high-risk cases. Payers control network design, QPA construction, claims-processing timelines, authorization requirements, and payment offer strategy. Regulators control benchmark methodology, filing rules, and IDR framework design. Hospitals and corporate practice entities control staffing, service-line structure, access conditions, and the terms under which physicians operate [7].

After the relevant payment construct has been identified, the next step is to test whether that construct is being used within its proper interpretive limits. Table 3 presents the five-filter construct-separation test, which asks who generated the number, how it was constructed, what it was designed to measure, whether it fits the clinical context, and what should not be inferred from it. This sequence is intended to prevent unsupported extrapolation from one reimbursement construct to another.

**Table 3:** Five-filter construct separation test. Proposed analytic framework for evaluating whether a reimbursement construct is being applied within its purpose, scope, and interpretive boundaries.

Filter	Question
1. Source and provenance	Who generated the number, and from what data source?
2. Construction method	How was the number calculated, selected, negotiated, or adjudicated?
3. Intended purpose	What was the number designed to measure or administer?
4. Clinical fit	Does the number reflect the urgency, complexity, and care environment at issue?
5. Interpretive boundary	What should not be inferred from this number?

Once the construct has been defined, accountability should be evaluated by identifying who controlled the operative variable that produced the disputed outcome. Table 4 presents the six-question variable-attribution test, which distinguishes authority, visibility, financial exposure, data control, bedside risk, and visible accountability. The test is intentionally neutral and may identify physician-controlled, payer-controlled, hospital-controlled, regulatory, administrative, corporate, or shared variables depending on the facts of the case.

**Table 4:** Six-question variable-attribution test. The framework is neutral and may identify physician, payer, hospital, regulatory, administrative, corporate, or shared control depending on the facts.

Question	Analytic Function
Who had authority?	Which party had power to approve, deny, delay, contract, benchmark, process, or restrict the service?
Who had visibility?	Which party was seen by the patient and likely to receive patient-facing attribution, whether or not they controlled the outcome?
Who had relevant financial exposure?	Which party bore financial consequences from the outcome, including delay, denial, underpayment, or shifted risk?
Who controlled the data?	Which party controlled claims data, contracted-rate data, denial data, authorization data, or adjudication data relevant to the dispute?
Who carried bedside risk?	Which party bore immediate clinical, ethical, reputational, or professional risk when care was needed?
Who absorbed visible accountability?	Which party became the face of the dispute, regardless of whether they controlled the underlying variable?

Because the framework is intended as an analytic method rather than an intuitive judgment exercise, reproducibility depends on reviewers applying the same sequence of questions in the same order. Construct separation should be completed before variable attribution, and each question should be answered independently using the available evidence rather than inferred from the final reimbursement outcome. When multiple reviewers disagree, the source of disagreement should be examined, including differences in construct classification, interpretation of operative control, available evidence, or ambiguity in the framework’s definitions. Future empirical studies should evaluate inter-rater agreement using standardized reviewer instructions and identical reimbursement scenarios.

Framework figures

**Figure 1:**
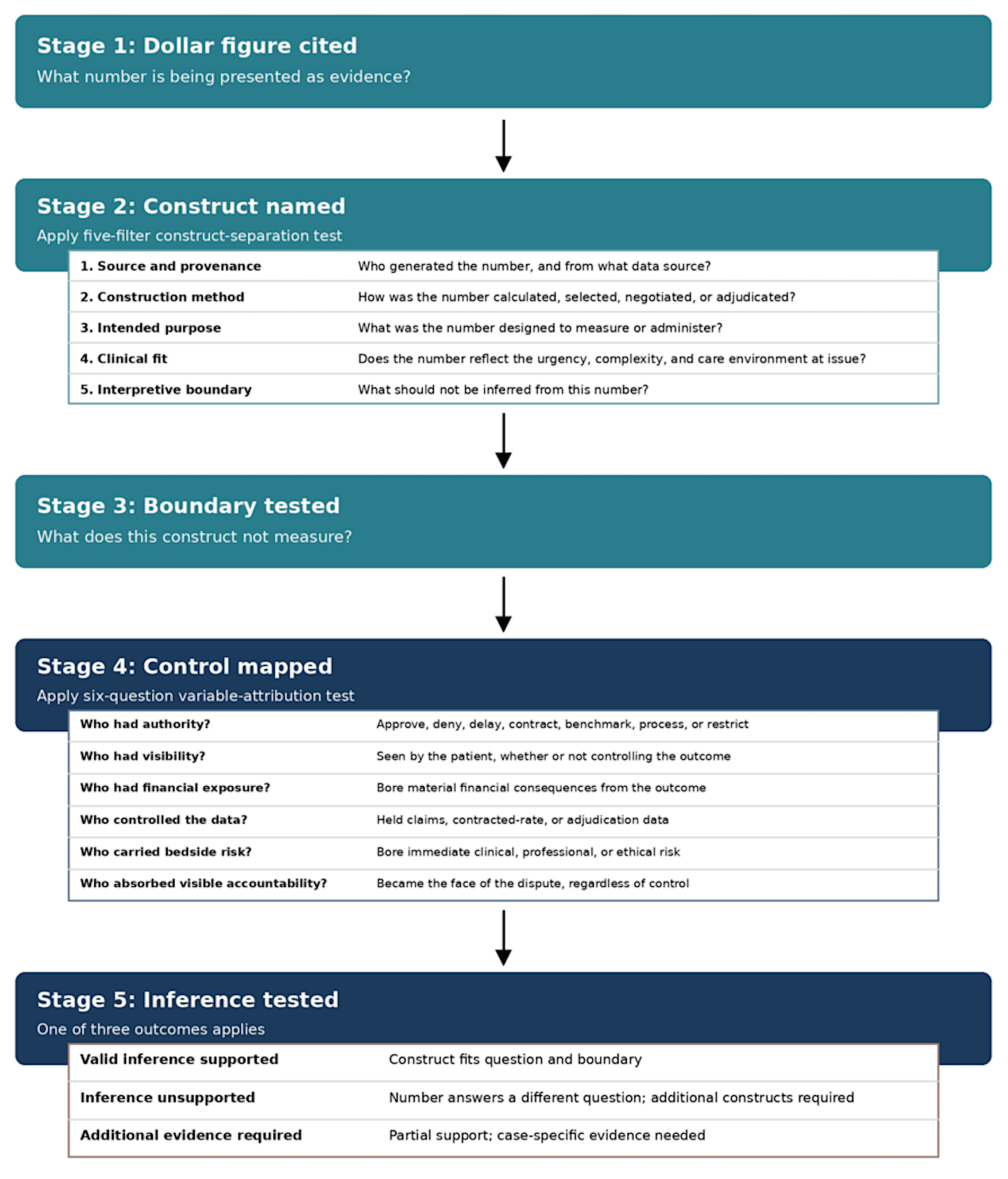
Construct Separation and Variable-Attribution Pathway This five-stage pathway illustrates the analytic sequence that should precede any policy inference drawn from a reimbursement figure. The evaluator first identifies the economic construct, then determines its source and construction method, defines its intended purpose and interpretive boundary, evaluates clinical fit, and finally maps control of the operative variables before assigning accountability. The pathway applies to physician charges, payer benchmarks, adjudicated outcomes, network-directory claims, and cost-containment assertions. Failure at any stage limits the validity of the resulting inference. This is an original conceptual schematic; no patient data were used.

Figure 1 illustrates the five-stage analytic sequence that should precede any policy inference drawn from a reimbursement figure. The pathway applies with equal force to physician charges, payer benchmarks, adjudicated outcomes, network-directory claims, and cost-containment savings assertions.

**Figure 2:**
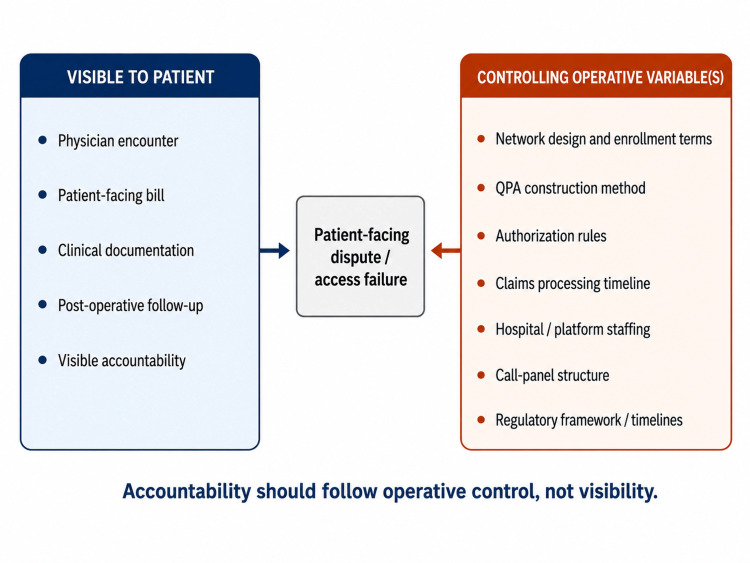
Visibility-Control Mismatch in Reimbursement and Access Disputes This schematic distinguishes the actors most visible to the patient from the actors or structures that may control the variables producing a reimbursement dispute or access failure. The patient-facing physician or hospital may be highly visible while having limited control over network design, benchmark construction, claims processing, authorization rules, or statutory dispute procedures. Conversely, visible clinicians may control treatment selection, documentation, coding, and case acceptance. The figure does not assign blame to any specific party; it demonstrates why accountability should follow operative control rather than visibility alone. This is an original conceptual schematic; no patient data were used.

Figure 2 illustrates the structural gap between the actor most visible to the patient and the actor or structures that controlled the operative variables producing a reimbursement dispute or access failure. The mismatch may favor any party depending on which variables were controlled by whom in the specific case.

Functional availability as the access-relevant construct

Network adequacy as currently measured constitutes its own form of benchmark-domain mismatch. A directory listing establishes that a specialist is contractually enrolled in a network. Federal network-adequacy standards include quantitative review concepts such as time-and-distance standards and appointment wait-time standards, but these measures do not necessarily establish whether a specialist is functionally available for the patients and conditions that make specialist access matter most [8,9].

Functional specialist availability is a distinct construct from directory presence. Directory adequacy is a count, whereas functional availability is a capability. The gap between them may be largest in high-acuity procedural care, emergency and after-hours coverage, revision and salvage cases, complex transfers, and care for geriatric, pediatric, or medically fragile populations [7-9].

Table 5 summarizes practical dimensions of functional availability, including appointment access rates, emergency-call participation, willingness to accept complex and revision cases, transfer acceptance rates, after-hours and weekend coverage, case-mix capability, response time for urgent referrals, and post-operative continuity. Directory-based standards that count enrolled specialists without measuring these dimensions may satisfy enrollment requirements while leaving unresolved whether meaningful functional access exists for the clinical scenarios at issue [8,9].

**Table 5:** Functional availability: dimensions, measurement indicators, and where directory presence fails. Proposed dimensions of functional specialist availability and examples of operational indicators beyond directory enrollment.

Dimension	What It Measures	Directory Proxy Fails When...
Appointment access	Whether new patients can obtain timely evaluation.	Listed specialist is not accepting new patients or has extended wait times.
Emergency-call participation	Whether specialist is available for urgent after-hours intervention.	Specialist has reduced or eliminated call coverage.
Complex and revision case acceptance	Whether specialist accepts high-acuity, revision, or salvage cases.	Specialist limits practice to lower-acuity elective cases.
Transfer acceptance	Whether specialist accepts transfers from other facilities or emergency departments.	Specialist or platform declines transfer workload.
After-hours and weekend coverage	Whether coverage is available outside standard business hours.	Coverage is nominal or redirected to emergency departments.
Pediatric / geriatric / salvage capability	Whether specialist has case-mix capability for vulnerable populations.	Subspecialty capacity is assumed from general network listing.
Post-operative continuity	Whether specialist maintains responsibility through recovery and complications.	Continuity is fragmented across employed or corporate structures.

These dimensions could be operationalized through prospectively measurable indicators, including the proportion of new-patient requests resulting in completed appointments, median wait time for routine and urgent consultation, percentage of emergency-call shifts covered, transfer acceptance and refusal rates, proportion of complex or revision referrals accepted, after-hours response time, percentage of cases redirected because of age or case-mix limitations, and completion rates for post-operative follow-up after complications. Measurement definitions, observation periods, denominator selection, and case-mix adjustment would require prospective validation before these indicators could be used for regulatory or comparative purposes.

Application to federal IDR

Federal IDR converts reimbursement disagreement into a structured comparison of offers within a statutory framework. Yet even in IDR, the visible offer spread is downstream of variables controlled at different points in the system: network design may determine whether the dispute arises; QPA construction shapes the administrative anchor; documentation quality shapes case-specific evidentiary strength; claims processing may reduce payment before IDR begins; and filing fees, timelines, and administrative steps alter the cost of pursuing the dispute [11,13].

Completed IDR outcomes are observable administrative results, not self-validating measures of fair value. Their interpretation depends on the statutory decision process, evidentiary inputs, selection effects in the completed dispute population, and the control variables that shaped the dispute before adjudication. The relevant policy point is not that adjudicated outcomes establish a preferred price, but that they cannot be interpreted without identifying the construct and process that produced them.

Delay in payment resolution affects both parties differently in terms of present-value exposure and capital timing. For physicians and practices, delayed payment may reduce present value, create financing pressure, and increase documentation burden. For payers, delayed payment may affect cash timing in ways that influence portfolio-level settlement behavior. Recognizing this asymmetry requires no inference of misconduct; it recognizes delay as an economically material variable.

The visibility-control mismatch can be clarified by mapping visible reimbursement or access problems to the variables that may have produced them. Table 6 provides examples of common payment and access issues, the visible object encountered by the patient or clinician, the potential controlling variables behind the issue, and the analytic question needed to determine whether accountability follows actual operative control rather than visibility alone. The examples in Table 6 are hypothetical policy scenarios constructed to demonstrate application of the framework; they are not extracted from identifiable disputes or used as empirical evidence of causation.

**Table 6:** Control map: visible payment issue, potential controlling variable, and key analytic question. Examples are illustrative and are designed to promote systematic analysis of operative control rather than reliance on visibility alone. MPFS = Medicare Physician Fee Schedule; QPA = qualifying payment amount; NSA = No Surprises Act; IDR = independent dispute resolution.

Payment Issue	Visible Object	Potential Controlling Variable	Key Analytic Question
Out-of-network dispute	Physician bill	Network design, contract terms, QPA construction	Was the network functionally adequate before the service was needed?
Delayed payment	Provider claim	Claims-processing rules, dispute timeline, filing requirements	Who controlled the delay, and who bore the time-value effect?
Benchmark reliance	QPA or MPFS rate	Benchmark source, construction method, statutory purpose	Does the benchmark measure the economic question being asked?
High-acuity surgical case	Procedure code	Clinical complexity not fully captured by code alone	Did the cited benchmark fit the clinical environment at issue?
Service-line access problem	Unavailable specialist	Network design, call-panel structure, hospital staffing	Was access measured by directory listing or functional availability?
Downstream complication cost	Index professional fee	Clinical risk trajectory, facility utilization, post-acute care	Was total episode cost measured, or was it merely inferred from the index fee?

Policy implications and safeguards

A reimbursement policy that acts on a number without identifying its construct, boundary, and controlling actor repeats the category error at scale. A potential implementation strategy would be to encourage construct labeling for every reimbursement figure used in policy analysis, public reporting, IDR submissions, or network-adequacy review: the source of the number, the method by which it was constructed, its intended administrative purpose, and the inference it can and cannot support should be stated before the number is used as evidence [1-6,10,11,13]. Public reporting and expenditure analysis should distinguish physician professional reimbursement from facility revenue, insurer administration, pharmaceutical spending, PBM intermediary activity, corporate overhead, and administrative expense before assigning cost-growth responsibility to any sector [1-6].

For Medicare and NSA-related policy, MPFS update mechanisms should be evaluated not only as budgetary instruments but also as determinants of clinical infrastructure, emergency availability, and specialty capacity in access-sensitive procedural and emergency-call-dependent settings [10,14,15]. QPA should be treated as a legally relevant administrative benchmark whose evidentiary significance depends on provenance, construction method, clinical fit, and case-specific statutory factors [11,13]. For network adequacy, regulators and payers should consider functional availability metrics alongside directory enrollment, including appointment wait times, emergency-call participation, transfer acceptance, complex-case acceptance, after-hours coverage, and post-operative continuity [7-9]. Cost-reduction measures should be evaluated at the episode and system level before savings are claimed because reductions in an index professional fee do not establish downstream savings unless downstream utilization, access effects, and site-of-service migration are measured [1,3,12].

The proposed framework has relevance across multiple policy and administrative audiences because each stakeholder may encounter different forms of construct substitution or attribution error. Table 7 summarizes how the framework may be applied by CMS/HHS, state regulators, legislators, hospitals, specialty societies, IDR entities, and cost-containment analysts. For each audience, the table identifies the construct error avoided and the measurement information needed to support valid policy interpretation.

**Table 7:** Policy relevance matrix: audience, construct error avoided, and measurement need. Summary of potential applications of the Visibility-Control framework across policy, regulatory, payer, provider, research, and administrative settings. MPFS = Medicare Physician Fee Schedule; QPA = qualifying payment amount; NSA = No Surprises Act; IDR = independent dispute resolution.

Audience	Construct Error Avoided	Measurement Need
CMS / HHS	Treating administrative rates as comprehensive economic value.	Benchmark construction, statutory purpose, and clinical-fit boundaries.
State regulators	Treating directory participation as real specialist access.	Functional availability: call coverage, transfer acceptance, case-mix capability, appointment access.
Legislators	Assuming visible professional-fee reductions equal system-level savings.	Episode-level cost decomposition including downstream utilization and access effects.
Hospitals	Conflating professional payment with downstream institutional exposure.	Readmission, reoperation, length of stay, transfer burden, post-acute utilization.
Specialty societies	Reducing complex procedural care to code-only comparisons.	Clinical complexity variables, functional availability burden, and downstream failure risk.
IDR entities	Substituting one dollar-denominated construct for another.	Evidence classification by source, construction method, intended purpose, and clinical fit.
Cost-containment analysts	Measuring savings at the index-fee level only.	Total episode cost, downstream utilization, access effects, and site-of-service migration.

Because the framework could be misinterpreted as a directional reimbursement argument, safeguards are needed to preserve its neutral analytic purpose. Table 8 summarizes anticipated critiques and corresponding manuscript safeguards. These safeguards emphasize that construct separation and variable attribution must be applied symmetrically to physician, payer, hospital, regulatory, and administrative variables, and that the framework is not intended to establish payment amounts, legal liability, causal proof, or access adequacy by itself.

**Table 8:** Anticipated critique and manuscript safeguards. Safeguards reinforce the framework as an analytic and interpretive tool rather than a payment standard, legal test, or liability assessment. MPFS = Medicare Physician Fee Schedule; QPA = qualifying payment amount; NSA = No Surprises Act; IDR = independent dispute resolution.

Likely Critique	Assessment	Safeguard
This is physician-fee advocacy.	Partially valid	Framework states construct separation is a validity requirement that may support lower payment when the provider-side construct is unsupported.
QPA is legally central under the NSA.	Valid	QPA legal relevance is explicitly acknowledged; the concern is applying it outside its construction domain.
Variable attribution always points at payers.	Valid	The test is bidirectional and expressly includes physician-controlled variables.
IDR outcomes look like settlement strategy.	Partially valid	Section frames adjudicated outcomes as construct-dependent observations, not a formula, floor, or ceiling.
No causal proof of access harm.	Valid	Access effects are framed as plausible mechanisms requiring empirical testing.
Coding and modifiers already capture complexity.	Partially valid	Framework asks whether the specific benchmark incorporated the clinically material variables documented in the case.
Adjudicated payments do not prove fair value.	Valid	Adjudicated payments are observed dispute outcomes, not normative price standards.
Billed charges may be inflated.	Valid	Billed charges are described as provider requests requiring independent support.
Denominator argument just minimizes physician costs.	Valid	Denominator discipline applies symmetrically to all sectors.

## Discussion

The principal contribution of this report is the distinction between visibility and control as separate analytic constructs in reimbursement-policy analysis. Payment amounts, benchmarks, directory listings, and dispute outcomes are often interpreted as though visibility alone establishes accountability. The proposed framework argues that valid policy analysis requires independent assessment of both construct identity and operative control. This distinction is consistent with the need to separate national expenditure categories, Medicare payment-administration mechanisms, NSA benchmark methodology, and IDR outcomes before drawing broader conclusions about value, access, or accountability [1-6,10,11,13-15].

The framework also emphasizes construct separation as a prerequisite for valid inference. Physician professional fees, Medicare payment rates, QPAs, billed charges, adjudicated payments, and total episode expenditures are related but distinct economic constructs. Each was developed for a specific administrative or operational purpose and may lose interpretive validity when applied outside its intended domain. A practical recommendation is that policy analyses and IDR submissions should identify the construct being cited, document its provenance, state its intended purpose, and explicitly describe what it does not measure before using it to support a reimbursement or cost-containment conclusion [1-6,10-13].

A second contribution is the concept of variable attribution. Accountability should follow control of the operative variable rather than default assignment to the actor most visible to patients or policymakers. Physicians, payers, hospitals, regulators, and corporate entities may each control different aspects of reimbursement, access, and care delivery [7,11,13]. Potential solutions include standardized control maps in reimbursement disputes, separate reporting of payer-controlled and provider-controlled variables, and documentation templates that distinguish clinical complexity from administrative benchmark construction. These steps would not determine the payment outcome by themselves, but they would reduce the risk that a visible bill, directory listing, or benchmark is treated as complete evidence of accountability.

The framework also suggests specific network-adequacy improvements. Directory participation should be supplemented with functional specialist availability measures, including appointment access, emergency-call participation, transfer acceptance, complex-case acceptance, after-hours coverage, case-mix capability, and post-operative continuity. Federal network-adequacy standards already incorporate quantitative concepts such as time-and-distance and appointment wait-time standards, which support the feasibility of moving beyond directory counts; however, procedural specialty access may require additional operational measures that capture emergency and complex-care capacity [8,9]. The framework can itself be misused if treated as a directional argument rather than an analytic sequence. Construct separation does not imply that physician charges are appropriate, that payer benchmarks are invalid, or that adjudicated payments define market value. Variable attribution does not imply that the least visible actor is responsible or that the most visible actor is blameless. Any use that skips classification, boundary analysis, control mapping, and inference testing repeats the category error the framework is intended to prevent [11,13].

Practical implementation may also be limited by stakeholder variability and inter-rater subjectivity. Different reviewers may classify the same reimbursement construct differently, assign shared control to different actors, or disagree about whether a benchmark adequately fits the clinical context. Application may also vary across specialties, geographic markets, contractual structures, and regulatory settings. Standardized definitions, reviewer training, structured scoring instructions, and testing with identical case scenarios will therefore be necessary before the framework can be used reliably for comparative, regulatory, or adjudicative purposes.

Limitations

This framework is conceptual and hypothesis-generating. It does not prove that professional-fee compression causes access limitations, determine appropriate payment in any individual dispute, or establish that any administrative benchmark is inaccurate. Access outcomes are multifactorial; geography, workforce supply, liability exposure, hospital resources, payer contracting, employment trends, consolidation, network design, and patient population characteristics may all contribute [7-9].

The discussion of adjudicated outcomes as construct-dependent observations is illustrative only. Adjudicated outcomes reflect selected completed disputes and should not be generalized as universal price standards, used to create an evidentiary floor, or treated as operational settlement benchmarks [11,13]. Variable attribution requires case-specific application; control differs across disputes, practice settings, geographic markets, contractual arrangements, and organizational structures.

Future research

Future empirical work should evaluate physician professional-fee share of total episode expenditures across specialties, settings, and levels of case complexity; compare MPFS rates, QPAs, contracted rates, billed charges, and adjudicated payments within matched cohorts; assess relationships between reimbursement levels and functional specialist availability; examine whether professional-fee compression is associated with consolidation or site-of-service migration; and determine whether reductions in index payments are offset by downstream utilization costs in complex-care trajectories [1-3,7-15].

Future validation should also test the framework’s reproducibility and practical effect. Independent reviewers should apply the five-filter construct-separation test and six-question variable-attribution test to identical reimbursement and access scenarios, with assessment of inter-rater agreement, sources of disagreement, and the effect of training or standardized instructions. Subsequent studies should determine whether use of the framework changes benchmark interpretation, accountability assignment, network-adequacy assessment, or reimbursement-policy decisions compared with unstructured review.

## Conclusions

Healthcare reimbursement analyses frequently compare payment figures that share a common monetary unit but represent fundamentally different economic constructs. Physician professional fees, facility payments, MPFS rates, QPAs, billed charges, adjudicated payments, and total episode costs are not interchangeable measures and should not be used as proxies for one another without careful attention to their source, purpose, and interpretive boundaries. The Visibility-Control framework proposes a structured approach to reducing construct drift and attribution error through construct separation, variable attribution, and functional availability assessment. By asking what is being measured, what the construct was designed to represent, what it omits, and who controlled the operative variable, the framework seeks to improve the validity of reimbursement-policy analysis, benchmark interpretation, network-adequacy evaluation, and accountability assessments. The framework is hypothesis-generating and has not yet been empirically validated for effectiveness, reproducibility, inter-rater agreement, or impact on reimbursement-policy decisions. Until such validation is performed, it should be viewed as an analytic and interpretive tool rather than a payment standard, access model, causal model, or reimbursement-determination instrument.
